# Process Evaluation of a Randomized Controlled Trial With a Mobile Health Intervention for Children With Obesity

**DOI:** 10.1177/30502225251348292

**Published:** 2025-06-25

**Authors:** Linnea Hedin, Maria Hagströmer, Claude Marcus, Pernilla Danielsson

**Affiliations:** 1 Department of Clinical Science, Intervention and Technology, Division of Pediatrics, Karolinska Institutet, Stockholm, Sweden; 2 Allied Health Professionals, Karolinska University Hospital, Stockholm, Sweden; 3 Department of Neurobiology, Care Sciences and Society, Division of Physiotherapy, Karolinska Institutet, Stockholm, Sweden; 4 Academic Primary Health Care Centre, Region Stockholm, Sweden; 5 Sophiahemmet University, Stockholm, Sweden

**Keywords:** attrition, mHealth intervention, Normalization Process Theory, pediatric obesity, recruitment, weight management

## Abstract

**Objectives:**

To explore barriers for recruitment, attrition, and mHealth usage in a Swedish randomized controlled trial (RCT) including a mobile health (mHealth) intervention for children 5 to 12 years (n = 79) in obesity treatment.

**Methods:**

A retrospective process evaluation was conducted using Normalization Process Theory (NPT). To answer the study objectives, data on staff- and parental mHealth experience and usage, staff working time, recruitment, and reasons for attrition were evaluated in relation to the NPT constructs.

**Results:**

Recruitment process was complex and trial enrollment during the summer decreased staff- and participant-engagement. Attrition was influenced by technical issues, lack of motivation, and disliking the mHealth intervention. The major barrier for mHealth usage was technical problems. Staff struggled understanding core intervention components and found the intervention time consuming.

**Conclusion:**

Based on our findings we suggest future enablers for sufficient recruitment, retention, and increased mHealth usage of value for conducting pediatric obesity trials.

## Introduction

To evaluate new interventions, randomized controlled trials (RCT) are considered the gold standard.^1^ However, for RCTs the recruitment of participants can be challenging, with approximately half of the trials reaching their recruitment target.^2-5^ Recruitment failure results in underpowered analyses with the consequence that a potential effect is not detected. Insufficient recruitment combined with high attrition, which is a severe issue in pediatric obesity management and studies,^6,7^ results in even greater power issues. Further, non-adherence and performance bias can constrain the ability to draw valid conclusions about intervention effects.^8^ Approximately 20% to 60% of conducted trials are not published several years after trial finalization, and trials with non-significant findings are less likely to be published.^9-15^ Reasons for the high proportion of non-publications might be multiple, for example, difficulties to get manuscript accepted for publication, unwillingness to share trial results, or experienced challenges with trial design and performance bias. Regardless, reporting findings for trials that could not be conducted as intended is important so that others could learn from the mistakes and avoid them in future trials. In addition, from an ethical standpoint, it is a concern that many children and their parents participate in unpublished clinical trials that may include additional physical appointments and blood sampling.^16^

Obesity in childhood is associated with increased risk of pre-mature death in adulthood^17,18^—already before 25 years of age.^19^ When assessing treatment results in children with obesity, body mass index (BMI) is not an optimal measure since it does not take the child’s growth pattern into account. Therefore, age- and sex-specific standard deviation score (SDS) of BMI has been developed to allow for detecting individual changes in obesity in the growing child together with the possibility to compare degree of obesity between children of different age and sex.^20^ A decrease in degree of obesity, that is, a decrease of BMI SDS of at least 0.25 units is associated with improvements of cardiometabolic risk factors.^21,22^ Therefore, a reduction of 0.25 BMI SDS is often seen as a clinically relevant treatment outcome. Between the years of 2016 to 2017, children in Sweden attending obesity treatment had a mean (standard deviation, SD) reduction in BMI SDS of 0.13 (0.38) units. Further, attrition had increased by 20% between 2009 and 2017; hence, pediatric obesity treatment in Sweden needs to be improved.^23^

Mobile health (mHealth) interventions, delivered by mobile applications or health monitoring devices, have the advantage of providing a greater accessibility and flexibility compared with traditional face-to-face appointments. Studies on children with overweight and obesity show favorable results for mHealth intervention compared with other treatment approaches,^24^ however, effects on weight related outcomes are relatively small and more effective mHealth interventions are needed.^25^ With this background we conducted a feasibility trial on an mHealth intervention, which was positively received by parents and clinical staff.^26^ To further evaluate the effectiveness of the mHealth intervention, a 1-year multi-center RCT was conducted. Seventy-nine children with obesity, 5 to 12 years, received either an mHealth intervention in addition to standard treatment (intervention) or standard treatment (control). The conducted RCT (ClinicalTrials.gov, ID: NCT03566771) turned out to be inconclusive due to several flaws; technical problems, insufficient recruitment, high attrition, and low mHealth usage. With an ambition to comprehend why the RCT failed to provide evaluable data, we conducted a process evaluation based on Normalization Process Theory (NPT).^27^ The aim was to understand the barriers in specific relation to recruitment, attrition, and mHealth usage.

## Methods

In this trial, we conducted a retrospectively planned process evaluation, based on NPT,^27^ of; (a) aspects of an RCT and (b) the mHealth intervention part of an RCT. To answer the study aim, a sample of questions within the NPT structure was selected to describe and evaluate the trial, the intervention, and the context. Initially, when conducting the outline of the RCT, a process evaluation was not planned for. Since we believe that understanding trial limitations is called for, we decided to conduct a process evaluation planned retrospectively. Hence, this process evaluation contains data collected and observed in the RCT which have been structured, analyzed, and presented using a theoretical framework to answer questions on barriers for recruitment, attrition, and mHealth usage.

### Normalization Process Theory

NPT is a model to evaluate implementation of complex interventions within the healthcare system. The theory focuses on the work individuals or groups do to make an intervention become a natural part of the clinical environment.^28-31^ NPT can also be used as a framework for designing and planning interventions and trials.^27^ The theory has 4 main components; *coherence*, focusing on meaning and sense-making of the intervention; *cognitive participation*, aiming to address engagement in individuals or groups; *collective action* that is, the work that is done to make the intervention happen; *reflexive monitoring*, aiming to target how the work is understood and valued by individuals or groups. These 4 components interact with each other and with the wider *context* for example, organizational structure and group processes.^27,29,30^

### Description of the RCT

#### Aim and Trial Design

The aim was to evaluate if an mHealth intervention in addition to standard treatment would give better results on degree of obesity compared with standard treatment. The trial was a parallel open-label multi-center RCT with 12 months duration, between the years of 2018 to 2019. As all pediatric health care in Sweden, study participation was free of charge. The participating clinics were users of the Swedish Childhood Obesity Treatment Register (BORIS)—a national quality register for children and adolescents in obesity treatment, where patient data was electronically registered by the staff at the clinic.^23^

#### Participants

The inclusion criteria were: (a) age 5 to 12 years, (b) obesity according to the International Obesity Task Force (IOTF),^20^ (c) no prior obesity treatment or obesity treatment the last 9 to 15 months with a decrease in BMI SDS ≤ 0.25 units, (d) parents speaking Swedish, (e) parents being able to use a smartphone, and (e) no pharmacological treatment affecting the obesity intervention. The exclusion criteria were (a) diagnosed or ongoing assessment of neuropsychiatric disorder, and (b) hypothalamic obesity.

By a digital randomization program used by the clinical staff at every site, the children were randomly allocated (1:1) to either mHealth intervention in combination with standard treatment (intervention) or to standard treatment (control). Each of the 10 pediatric clinics agreed to enroll a unique number of participants depending on the size of the clinic. Of the participants at each clinic, 50% should have no previous treatment and 50% previous treatment. The randomization was stratified with 2 blocks (no previous treatment and previous treatment) at each clinic.

#### Standard Treatment

The control- and intervention group received standard treatment, that followed the routine at each pediatric clinic, aimed to reduce the degree of obesity by improving dietary habits and increasing physical activity (PA). No other treatment options, for example, pharmacological treatment, were available. All participants had follow-up appointments with the clinical staff at least every third month according to the study protocol that is, at 3-, 9-, and 12-months post-randomization. Additional appointments could be scheduled if needed.

#### The mHealth Intervention

**Figure 1.**
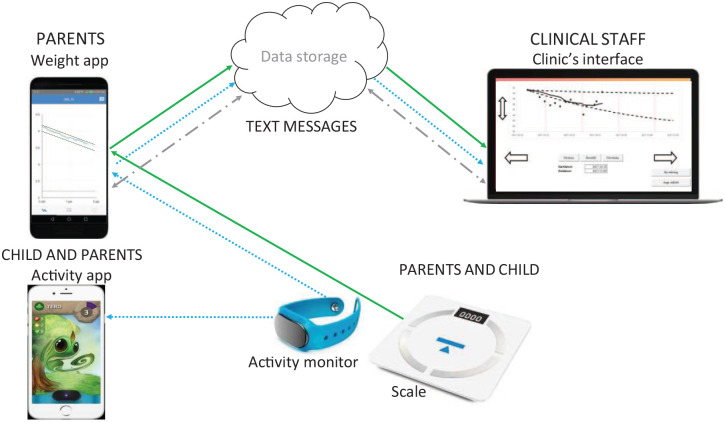
Illustration of the mHealth intervention. PARENTS AND CHILD: The child measured weight daily on a scale that did not display weight. The activity monitor was used by the child and time in physical activity (PA) was presented in the gamified Lifee app. PARENTS: The child’s weight was presented in the Provement app as body mass index standard deviation score (BMI SDS) and presented in relation to a weight loss target curve. The Provement app also displayed time spent in PA, and the app was used for communication with staff via text messages. CLINICAL STAFF: The child’s weight, BMI SDS, was presented on the clinic’s interface in relation to a weight loss target curve. The interface presented child’s time spent in PA, and was used for communication with parents, via text messages. Solid lines illustrate data transfer for measured weight. Dotted lines illustrate data transfer for time in PA. Dashed lines illustrate data transfer for text messages.

The intervention, called Provement, (Figure 1) was developed by Health Support Sweden AB (Stockholm Sweden) and consisted of a mobile application (app) for parental use and of a clinic’s interface to be used by staff. The app was a protype, compatible with Android and iOS, and not commercially available. The children took daily weight measurements at home and weight data was transferred from the scale to the mobile app via Bluetooth, and to the clinic’s interface through a digital cloud server. Weight outcomes were not shown on the scales, instead a weight loss target curve, based on mean value of BMI SDS the last 5 days, was presented graphically in the app. The same information was shown on the clinic’s interface together with more specific data on weight in kg and BMI SDS. The weight loss target curve had an upper and a lower BMI SDS limit and the slope of the curve was based on estimated growth for the following 3 months together with degree of obesity. Since growth velocity in children with obesity is different from children with normal weight, the growth estimation for the weight loss target curves was based on data from the BORIS register, including specific information on growth in children with obesity. At every clinic appointment (baseline and follow-up) the staff added the child’s weight and height to the system and a new weight loss target curve for the following 3 months was automatically generated by the system. In general, the range of the curve was set to −0.15 to −0.35 BMI SDS. Between clinic appointments, a child’s current BMI SDS values were based on data from weight assessments at home together with the longitudinal growth estimation. Moreover, the app could be used by parents to communicate with staff via text messages, and the staff used the clinic’s interface for digital communication with parents.

An additional intervention feature, aimed to increase motivation for PA, was a commercially available app, Lifee Spirits (Lifee AB, Norrköping, Sweden) that included of a wrist-worn activity monitor and app gamification. The activity monitor collected data on time spent in PA which was transferred to the gamified app via Bluetooth. Gamification included that rewards were generated, in terms of collecting diamonds of different values, when time spent in PA increased. An alteration from our previous feasibility trial^26^ was that every time weight was measured at home, the Provement app collected PA data from the activity monitor, resulting in a graphic presentation of PA levels in the Provement app seen by parents and on the clinic’s interface seen by staff. Parents were instructed to measure their child’s weight daily and that the activity monitor should be worn on the child’s non-dominant wrist. Moreover, parents were encouraged to send messages to staff whenever they wanted support. The staff were instructed to keep track of the participant’s weight related outcomes on the clinic’s interface at least weekly and to provide feedback to parents through sending messages.

The scales for self-weighing, the Provement app, the interface and data storage were provided by Health Support Sweden AB (Stockholm, Sweden). The activity monitor and gamified app were provided by Lifee AB (Norrköping, Sweden) via Health Support Sweden AB (Stockholm, Sweden).

#### Sample Size Calculation

According to the sample size calculation, for the RCT that this process evaluation is based on, 42 individuals were required in each group (intervention vs control) to detect a difference between the intervention and the control group in BMI SDS of 0.25 units with 80% power at the end of the study. With an estimated drop-out rate of 30%, 60 children in each group were required. Estimated standard deviation was set to 0.4. No additional sample size calculation was made for this retrospectively planned process evaluation.

### NPT Analysis

Descriptive data on background characteristics, attrition, recruitment, and change in BMI SDS are presented for the intervention- and the control group.

Selected questions for defining the context, and for analyzing the 4 NPT components for the trial and the intervention are presented in Textbox 1 and Table 1. Participants and user groups were defined as both clinical staff and parents/children. End users were defined as parents/children. Except for comparison of required working time between the control- and intervention group, the NPT analysis included parents/children from the intervention arm of the RCT. The NPT analysis included the following data:

- Staff’s mHealth experience (web-based questions at 3, 6, and 12 months)
- Parental mHealth experience (web-based questions at 3, 6, and 12 months)
- mHealth usage for parents/children and staff (weight- and message frequency, logging into the clinic’s interface)
- Recruitment (number of clinics and children)
- Self-reported working time from staff
- Attrition and reasons documented by staff
- Researchers’ comments

**Textbox 1.** Questions for defining the context based on Murray et al.^27^

1. Which staff groups were affected by the intervention? 2. What was their working environment like? 3. Were there any changes in their working environment during the trial? 4. What were the consultations like? 5. What systems were already in place at the clinics? 6. How well did trial procedures interact with these systems? 7. Did any major changes to the trial context occur during the trial period? 8. Timing of patient recruitment? 9. Timing of data collection? 10. What end users were affected by the intervention? 11. What could the end users’ environment be like?

**Table 1.** Questions Used for Performing the NPT Analysis.

NPT components	Trial—NPT questions	Intervention—NPT questions
Coherence^a^		
	Was the trial easy to describe?	Was the intervention easy to describe?
		Did the intervention have a clear purpose for all relevant participants?
		What benefits did the intervention bring and to whom?
		Did the intervention fit with the overall goals and activity of the organization?
		Was the intervention clearly distinct from other interventions?
Cognitive participation^b^		
	Were target user groups prepared to invest time and energy in the trial?	Were target user groups likely to think that the intervention was a good idea?
		Were target user groups prepared to invest time and energy in the intervention?
Collective action^c^		
	Did the trial procedure promote or impede the work of user groups?	Did the intervention promote or impede the work of user groups?
	Did participation in the trial require extensive training for staff involved?	What effect did the intervention have on consultations?
Reflexive monitoring^d^		
	Could participants contribute feedback about the trial once it was ongoing?	How did users perceive the intervention once it had been in use for a while?

a Coherence, meaning and sense-making.

b Cognitive participation, commitment and engagement.

c Collective action, work done by participants to make the intervention/trial happen.

d Reflexive monitoring, participants reflect and appraise the intervention/trial.

The questionnaires were specifically compiled for the RCT and contained mostly closed-ended, but a few open-ended questions regarding advantages and disadvantages with the mHealth approach. Weight- and message frequency were automatically registered on the interface and log-in frequency was self-reported from staff. Required working time, including documentation, in terms of time for physical appointments, phone calls, and sent messages were documented on the clinic’s interface by the staff throughout the whole trial. Comments from the authors were added where collected data could not be used to address the questions for NPT analysis.

Weekly number of weights for each month and required working time in minutes are presented as median and interquartile range (IQR). Weight frequency is presented for those remaining in the trial, that is, data from dropouts were included until attrition occurred. Required working time for staff, intervention versus control, includes participants completing the whole trial and were analyzed with the Mann Whitney U-test (IBM SPSS Armonk, NY, USA, version 28). A *P*-value < .05 was considered as statistically significant.

To identify barriers, all data was mapped to one of the questions used for the NPT analysis (Table 1) where it was most suited to provide a response to the NPT question. The mapping of the data was led by the first author with support from the last author. Possible reasons for barriers were based on responses from the questionnaires together with experiences from conducting the trial (discussion between first and last author). Future enablers are based on reflections from all authors together with existing literature on related topics.

### Ethical Approval and Informed Consent

The study was approved by the Ethical committee in Stockholm, no. 2018/478-31/2 and registered at ClinicalTrials.gov, ID: NCT03566771. An amendment from the original study protocol was made, extending the study from lasting 6 to 12 months (approval no. 2018/1759-32). If a family met the inclusion criteria and was interested in participation, the clinical staff informed the parents and child about the study and obtained their written informed consent together with written assent from the child.

## Results

### Pitfalls of the RCT

**Figure 2.**
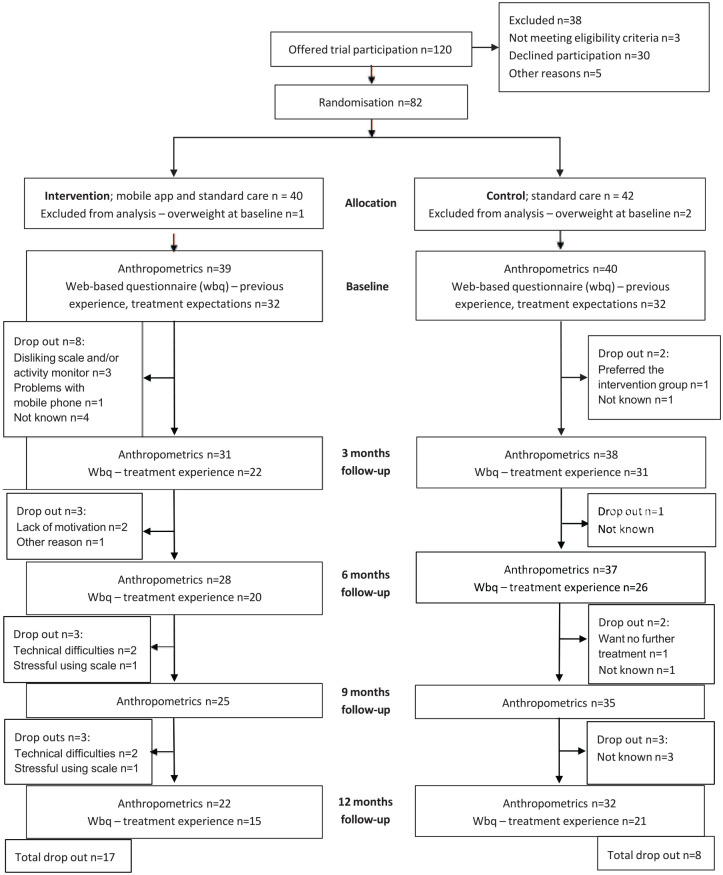
CONSORT participant flowchart including process evaluation aspects.

Recruitment was highly deficient (66% of the needed number according to the power calculation); the attrition rate was more than twice as high in the intervention group compared with the control group (44% vs 20%); and the intervention group used the mHealth intervention, in terms of self-weighing, less than intended. A CONSORT flowchart is presented in Figure 2, including process evaluation aspects in terms of recruitment, attrition, available anthropometrics data, and response rate for web-based questionnaires. Baseline characteristics, recruitment- and attrition frequency, mHealth usage, and change in BMI SDS are presented in Supplemental Tables 1 and 2.

### The Context

Of the 17 contacted clinics, 11 agreed to be a part of the RCT. Before trial initiation, one clinic chose to withdraw due to long-term sick leave. The 10 participating clinics were located from the very south to the north of Sweden and all had used BORIS for at least 2 years. All clinics had behavioral treatment as an approach according to their own routine and no clinics were involved in trials or using other concepts of behavioral treatment. No clinic or none of the staff in the trial solely consulted children with obesity, but also with other conditions for example, asthma and allergy.

There was a responsible medical doctor at each clinic, however, 14 staff members with other professions (pediatric nurse, dietician, physiotherapist, assistant nurse) were mainly in charge of participants in the trial. At 4/10 clinics, 2 staff members were actively involved in the trial. The work was either organized as working in pairs, including meetings and discussions about trial participants, or as individual work. At the 6 remaining clinics 1 staff member was actively involved in the trial. At 2 of these clinics the staff members terminated their employment during the trial, and were replaced by new staff, without the same training of the intervention and trial procedures.

Before trial initiation, all clinics used a medical records system and BORIS. The RCT required an additional electronic system in terms of the clinic’s interface. Every appointment was registered in BORIS by either the staff involved in the trial or by a coordinator at the clinic. The staff were informed to use BORIS in the recruitment process to determine if potential participants fulfilled the inclusion criteria.

Each clinic agreed to recruit an individual number of participants (range 8-20), and 3/10 clinics were able to enroll the agreed quantity. At 4/10 clinics, the enrollment was ≤50% of the anticipated number of participants. Recruitment commenced in April 2018 and was closed in Sep 2018, and 60/79 participants were included before July 2018. In total 120 children were asked to participate in the trial, of which 38 declined. Provided reasons for not participating (n = 23) were; travel plans or not willing during the summer (n = 5); consent from only 1 parent (n = 2); not interested in additional weight management support (n = 8); the trial was considered as too complex or as too much work (n = 3); decreased BMI and not fulfilling inclusion criteria (n = 1); skepticism toward measuring weight daily (n = 1); parents or child not interested (n = 2); and parent felt that the child was too young for participation (n = 1). Three children were enrolled in the trial despite not having obesity and were therefore excluded from analyses.

Depending on date of inclusion, follow-up appointments were planned during the summer or Christmas holiday for some participants. The first 3 months of the trial overlapped with summer vacation for both staff and parents. To have the ability to assess how the intervention was received by users in an everyday context, the trial was extended from 6 to 12 months.

The child and 1 or 2 parents attended all appointments together. Digital communication occurred solely between staff and parents. The staff needed to adjust treatment based on family situation for example, separated parents might need to work with different lifestyle modifications.

### NPT Components

#### Evaluation of the RCT

*Coherence* among staff was negatively affected by the inclusion criterion of previous obesity treatment with a change in BMI SDS ≤0.25 units. Several staff members struggled finding eligible participants based on this criterion and the need to use 2 digital systems (BORIS and the medical records system) was time consuming—which affected *collective action* negatively. The high attrition rate shows that c*ognitive participation* among parents and children was low. Reported reasons for attrition were technical issues, disliking the activity monitor and/or scale, lack of motivation, and that self-weighing was experienced as stressful. Trial enrollment before the summer holiday resulted in lower recruitment and might have affected the engagement during the first 3 months negatively. Further, c*ognitive participation* among staff members decreased over time, which might be related to the high attrition rate, technical difficulties, and the fact that the trial was extended from 6 to 12 months. No major adaptations to the trial procedure could be made based on feedback from participants, which might have been a barrier for *reflexive monitoring.*

#### Evaluation of the mHealth Intervention Within the RCT

In the NPT analysis of the intervention part of the RCT, technical issues were highlighted as a major problem for all NPT components. In total, parents reported 58 written comments on app-difficulties throughout the trial, of which 42 addressed technical issues and design problems with the activity monitor. The most frequent topics were that the activity monitor stopped working and had poor battery life, the wrist band was uncomfortable, problems with data transfer from the activity monitor and from the scale to the Provement app, and a need of logging in and out again to access the Provement app.

A core component of the intervention was the weight loss target curve, which illustrated the treatment goal. Therefore, it was a major barrier for *coherence* that half of the staff did not find the treatment goal clear and that some struggled to understand the target curves. This barrier could be related to the layout, technical issues, and that the curves were based on BMI SDS. High variation in message frequency, decreased activity on the clinic’s interface, and low weight frequency showed lack of *cognitive participation*. Weight frequency was low from the start of the trial and decreased further over time. It is possible that staff and parents focused more on the PA part of the intervention, and that daily weighing became secondary. Another reason might be that self-weighing was experienced as difficult for some participants. Decreased engagement, high attrition, and being responsible for few participants in the intervention group are possible reasons why the staff used the interface less over time. Further, the high variation in message frequency could be connected to the fact that some parents rarely answered text messages from staff.

Using the clinic’s interface was time-consuming for staff which decreased *collective action.* Although median required working time for appointments, phone calls, and documentation was similar between the control- and intervention group (*P* = .307), when sent messages were included the working time (min) was higher for the intervention—than the control group, median (IQR) 415 (203) versus 240 (81) min, *P* < .001 (Supplemental Table 4).

Another barrier was that we do not know how the staff used the interface during consultations. To minimize interference with each clinic’s routine of obesity treatment, guidance to staff in the consultation was deliberately not a part of the intervention. NPT analysis of *reflexive monitoring* showed that technical issues were major barriers for both staff and parents, resulting in increased time consumption and frustration. Further, it became harder to perform self-weighing and use the activity monitor over time. Besides technical problems, this may be related to the fact that the treatment outcomes did not meet the families’ expectations and that there were no updates in the game connected to the activity monitor.

Barriers with possible reasons, based on the NPT analyses, are presented in Tables 2 and 3, together with future enablers which are based on the authors’ reflections and existing literature (further elaborated in the discussion). Responses to questions for each NPT component are presented in Supplemental Tables 3 and 4.

**Table 2.** NPT Analysis of the Trial—Identified Barriers, With Possible Reasons, for Recruitment, Attrition and mHealth Usage.

Barriers by NPT components	Possible reasons^a^	Future enablers^b^
Coherence		
*Recruitment*: Complex inclusion criterion	BMI SDS not commonly used in the clinical setting	Broaden eligibility criteria
Cognitive participation		
*mHealth usage*: Low engagement from the start of the study	Summer holiday	The first 3 months should not overlap with longer holidays
*Attrition & mHealth usage*: Technical issues	Activity monitor with poor quality Combination of 2 mHealth systems	Avoid integration of 2 mHealth systems if they have limited maturity
*Attrition & mHealth usage*: Disliking scale and/or activity monitor	Technical issues Stressful measuring weights Lack of motivation	Prevent technical issues Discussions about self-weighing as a part of the intervention
*mHealth usage*: Lower engagement from staff over time	High attrition rate Trial extended from 6 to 12 months Technical issues, time consuming Few participants at each clinic; difficult to create a routine toward trial procedures	Create strategies to reduce attrition Avoid major changes to the study protocol during the trial Avoid very small populations at each clinic
Collective action		
*Recruitment & mHealth usage*: Staff used several digital systems for the trial	Time consuming	Avoid multiple digital systems or enable direct data transfer Researchers responsible for trial enrollment
*Recruitment* just before and during the summer	Staff and families on vacation	Avoid trial enrollment during longer holidays
Reflexive monitoring		
*mHealth usage*: The study design did not enable adaptations during the study period	Ongoing trial made it difficult to make adaptations	Test the interventions in a real-world clinic to make adaptations possible

a Possible reason for barriers.

b Future enablers stem from authors’ reflections and existing literature and are further elaborated in the discussion.

**Table 3.** NPT Analysis of the mHealth Intervention Within the Trial—Identified Barriers With Possible Reasons.

Barriers by NPT components	Possible reasons^a^	Future enablers^b^
Coherence		
*mHealth usage*: Half of the staff did not find treatment goal clear, some struggled to understand the weight loss target curves	Layout problems Technical issues Not familiar with BMI SDS^c^ Not enough training	Improve layout Prevent technical issues More education to staff about treatment outcomes (BMI SDS)
Cognitive participation		
*mHealth usage*: Message frequency varied widely for parents and for staff	High attrition Some parents not responding to staff Some staff rarely logged into the clinic’s interface Some parents and staff did not see the value with messages	Structured plan for: How message frequency can be individualized How to act if parents are not replying to messages
*mHealth usage*: Low weight frequency from the start of the study	Daily weights did not make sense to all participants Staff and families focused more on the PA part of the intervention Weighings were stressful to child and/or parents Technical issues	Educate staff and parents about: Why self-monitoring is important Decreased risk of eating disorders from structured obesity treatment That PA is important for other health aspects but has little effect on weight Guide staff to: Ask parents and child about experience from self-weighing Communicate with parents and/or child who experience stress during weighing Prevent technical issues
*mHealth usage*: Staff’s activity on the clinic’s interface decreased over time	Responsible for few participants—hard to establish routine usage High attrition; responsible for participants in control group only	Increase the number of participants at each clinic Several active staff members at each clinic to increase engagement
Collective action		
*mHealth usage*: Using the clinic’s interface was time consuming for staff and required working time was higher for the intervention group	Technical issues Parents not responding to messages Only one active clinician from 6/10 clinics	Prevent technical issues Several active staff members at each clinic to lower the workload
*mHealth usage*: We do not know how the staff used the interface during consultations	Guiding staff in the consultation was not a part of the design of the intervention	Built-in guidance to staff on the clinic’s interface regarding what the next step in the consultation should be
Reflexive monitoring		
*Attrition & mHealth usage*: Technical issues were seen as a major problem for parents and staff	Time consuming—the staff were contacted by the parents mostly about technical difficulties	Prevent technical issues A technical support function, which parents and staff can contact directly, is adviced
*mHealth usage*: It was harder to perform weighings over time	The treatment results did not meet the parents’ and/or child’s expectations Technical issues	Guide staff on self-weighing (see enabler, cognitive participation) To set realistic expectations, educate staff and parents about what treatment effects to expect from PA vs dietary changes Prevent technical issues
*mHealth usage*: Interest for the activity monitor decreased over time	Technical issues No updates in the game Uncomfortable to wear	Prevent technical issues Frequently update game or exclude activity monitor from intervention

a Possible reasons for barriers.

b Future enablers stem from authors’ reflections and existing literature and are further elaborated in the discussion.

c BMI SDS was the primary outcome measure in the RCT.

## Discussion

To our knowledge, this is the first process evaluation of an RCT including an mHealth intervention in young children with obesity. We identified several barriers for recruitment, attrition and mHealth usage and the findings together with future enablers are elaborated in the following sections.

### Recruitment

In our previous feasibility trial of the same mHealth intervention as analyzed in this process evaluation, children with no treatment the last 6 months were enrolled.^26^ To improve external validity, we decided to extend the inclusion criteria and to enroll participants with previous treatment if certain demands were fulfilled. However, the added criterion was found to be overly complex and to limit the efficiency of recruitment. Recruitment strategies, suggested by Foy et al,^32^ are to broaden the inclusion criteria and that research workers, instead of clinical staff, are responsible for recruitment. Both suggestions might have been valuable approaches for the evaluated RCT, to increase trial enrollment and to lower required working time for the clinical staff. However, in large clinical multi-center trials it may not be feasible to solely involve research workers in the recruitment process. Another time saving suggestion for future trials is to simplify identification of eligible study individuals.

In a systematic review of 37 RCTs it was found that recruitment rates increased when raising awareness about the health condition through a questionnaire or a video.^33^ In our process evaluation several children or parents declined trial participation because of little interest in additional support. It is possible that these families were not motivated, however, applying recruitment strategies to increase awareness about obesity could have affected their decision not to participate. Although timing of recruitment is not generally a highlighted recruitment strategy it can influence the recruitment effect,^27^ which was found in the evaluated RCT. We suggest that trial enrollment just before or during the summer or long holidays should be avoided.

In a recent systematic review of 26 pediatric trials including obesity-, nutrition-, and physical activity interventions, parents found that the major barriers for trial participation were time constraints and complex trial- and consent information.^34^ Therefore, it seems important to create study designs that lower time costs for parents, for example, by limiting physical appointments to reduce travel time, and to closely consider the number of activities required by parents in the trial. Additionally, creative ways of providing the parents and children with trial information may be necessary.

### Attrition and mHealth Usage

In RCTs, participants randomized to the intervention group are generally content with their allocation and participants in the control group are more likely disappointed, which is a potential source of performance bias.^8,35^ In our evaluated RCT it is likely that participants randomized to the intervention group initially were more satisfied with their allocation. However, it is highly possible that repeated technical issues with the mHealth intervention quickly resulted in a shift from satisfaction to disappointment, which increased attrition and decreased mHealth usage. In accordance with our findings, Browne et al ^36^ showed that technical problems with mHealth apps for pediatric obesity management was a major driver for high attrition and low engagement in the intervention group. In our feasibility trial of the evaluated mHealth intervention^26^ parents and staff experienced technical problems, but to a much lesser extent. Despite thorough testing of the intervention before the RCT commenced, we underestimated the need for further actions. Additionally, a technical support function should have been implemented before the trial was initiated.

High attrition in pediatric obesity treatment is a well-known problem.^6,7^ In clinical pediatric obesity trials, approximately one third of all studies have a high risk of attrition bias.^37^ Logistic barriers have been suggested as the most common reason for attrition,^38-40^ but also dissatisfaction with the treatment approach^41^ and different treatment expectations between parents, children, and staff^40^—aspects that could be reflective of the reported lack of motivation as a reason for attrition in our trial. In adults, strategies that successfully decrease attrition includes financial incentives, multicomponent treatment approaches, and self-monitoring, whereas face-to-face counseling have not been found effective.^42^ These strategies were not evaluated in a recent systematic review of 6 trials on reducing attrition in pediatric obesity treatment. Instead orientational sessions, motivational interviewing, and text messages were studied, and provided inconclusive results about potential effects on attrition.^43^ Digital interventions have the potential to reduce logistic barriers, and the mHealth intervention in this process evaluation also involves self-monitoring. It is our strong belief that attrition would have been lower in this trial if technical issues were reduced, however, further evaluation is required to substantiate this assumption.

In our process evaluation we found that some participants experienced difficulties or stress with self-weighing, however, we do not know which aspects of self-weighing were considered challenging. Since a bidirectional relationship has been found between frequency of self-monitoring and weight loss,^44^ monitoring weight in the studied population may have felt more difficult if the treatment goal was not met. Further, considering recent suggestions that weight-focused public health interventions for children may be harmful,^45^ it is not unlikely that some of the parents in our trial were worried about psychological negative side effects for their child from self-weighing. To our knowledge, no trials have evaluated potential negative effects from self-weighing in small children. Since measuring weight in exact terms, for example, in kilograms, is not an intuitive measure of change in degree of obesity in the growing child, many trials for children with obesity do not use self-weighing as a part of their intervention. In adults and young adults, systematic reviews have shown that interventions including self-monitoring of weight are not associated with any negative psychological health outcomes.^46,47^ In fact, weight management including self-weighing is associated with greater BMI reduction, improved body related attitudes, and increased health related quality of life.^48,49^ Therefore, self-monitoring of weight, or weight in relation to growth for children, appears to be an important aspect of obesity treatment. In addition, several recent systematic reviews have found that pediatric obesity treatment is associated with decreased risk of eating disorders and symptoms,^50-52^ and it is important to ensure that clinical staff and parents are aware of this. A future enabler for mHealth interventions in pediatric obesity may include education to staff and parents about self-weighing, as well as treatment and eating disorders.

A barrier found in this process evaluation was that staff struggled to understand the treatment goal and the weight loss target curve. Interestingly, almost all parents who responded to questionnaires did not experience these problems. Although mHealth usage among participants was low, it is possible that parents used the app more than staff used the clinic’s interface and therefore understood the treatment goal better. At some clinics, where work was divided between 2 staff members, it is further possible that some staff were mainly in charge of participants in the control group. Moreover, for all mHealth interventions, we believe it is important that the layout of for example, graphs is intuitive for both parents and staff.

The high variation in message frequency could be related to technical issues and low engagement from some parents and staff. Previous studies have found that the response rate from parents can be low,^26,53^ which in our study may have resulted in some staff sending fewer messages. There is no clear answer to how text messages should be used, however, several studies have found personalized content, frequency, and timing of delivery as important features.^54-56^ Therefore, a future enabler for mHealth interventions could be to include a structured plan for individualized message preferences.

In the beginning of the evaluated trial, parents and children were mostly excited about the gamified activity monitor, which might have led to a higher focus on PA in contrast to other important factors in behavioral change, for example, dietary habits and self-monitoring of weight. A combination of dietary changes and increased PA are recommended to be included in pediatric obesity treatment.^57^ Although PA has a positive impact on several obesity related co-morbidities,^58^ the effect on weight change is marginal.^59^ Therefore, it is highly important that clinical staff have this knowledge and share it with the parents to manage expectations on treatment effects. Future enablers could be to use an activity monitor of higher quality and to add frequent updates of the game in the activity app. Since the participants seemingly focused more on the activity monitor than the daily weight measurements, another possibility would be to discard the activity monitor in future trials. Technical problems occurred for both apps, but primarily, the commercially available PA device (no longer on the market) did not function properly on its own or together with the app tracking change in degree of obesity. Therefore, it is likely that 2 mHealth tools with limited technical maturity should not be used simultaneously in clinical trials. Failure of one app will have negative impact on the other, which makes the evaluation of both tools combined unreliable.

In our process evaluation we found that staff engagement decreased during the trial. Technical problems most likely affected engagement negatively, as described elsewhere.^60,61^ Another possible reason for decreased staff engagement is that only 1 or 2 staff members at each clinic were actively involved in the trial. Other studies have found that encouragement among co-workers is important for engagement, whereas lack of time is a major barrier that can result in stress and frustration.^60,61^ We found that the intervention arm of the evaluated RCT required more working time than the control group, which is in line with previous findings.^62^ Since pediatric obesity treatment in general lack treatment intensity^63^ it is expected that interventions aiming to increase the intensity will require more working time in comparison to standard treatment. Several active staff members at each clinic and preventing technical issues could potentially decrease the workload and increase engagement.

According to the NPT model,^27^ an RCT should preferably be preceded by analysis of potential issues with the study design and the intervention. In this process analysis several weaknesses with the study design and the intervention were found; some that could have been predicted and others that most likely would have been difficult to foresee. Based on our findings it seems obvious that the study design needs to be improved and that the mHealth intervention should not be tested again without major adjustments. Since the evaluated RCT commenced, the mHealth intervention has gone through extensive adjustments regarding layout and technical functionality. Additionally, the mHealth intervention (which is developed, upgraded and now commercially available and named Evira) solely focus on 1 digital component, in terms of the app including relative weight change and family–staff communication, without any evaluation of physical activity. The adjusted intervention has been evaluated at a pediatric obesity clinic in Stockholm and the results after 1 year were significantly better compared to a matched control group.^64^ To evaluate whether these results remain in a more general obesity population an international multi-center RCT is planned (ClinicalTrials.gov, ID: NCT04917601).

### Strengths and Limitations

A limitation of this study is that we did not have access to qualitative data. From the conducted feasibility trial^26^ we have knowledge about parents’ and staff’s experience, however, new barriers were found in the present study. Therefore, interviews with parents, children, and clinical staff would add important information about all evaluated aspects and should be included in future trials. Another limitation is that this process evaluation was planned in retrospect. Hence, no specific power calculation was conducted for the process evaluation and the collected data did not provide as in-depth results as if the data collection would have been adapted to the process related objectives. Nevertheless, we believe that results from this study provide valuable information for conducting future studies in pediatric obesity populations. A strength of this study is the additional knowledge about recurrent problems in pediatric obesity trials. By evaluating and sharing our experienced issues with the conducted RCT it is our hope and belief that other trials will be able to avoid several barriers resulting in high attrition, as well as low recruitment and low mHealth usage.

## Conclusions

This study highlights barriers and suggests future enablers regarding recruitment, attrition and mHealth usage based on an RCT including an mHealth intervention for children with obesity. To enable sufficient recruitment, researchers should strive to use wider inclusion criteria, avoid enrollment during long holidays, and limit administrative tasks for staff. For study retention and increased mHealth usage, preventing technical issues and providing real-time technical support seems vital. Further, attrition and mHealth usage may be improved by additional education to staff and parents about treatment and eating disorders, as well as what treatment effects to expect from physical activity versus dietary changes. Therefore, it is important for policymakers and healthcare providers to promote educational opportunities for staff working in pediatric obesity treatment. Additionally, it is likely that several active staff members are important for engagement and limited workload, and thereby mHealth usage can be improved. Finally, mHealth interventions are promising options to increase the treatment intensity without requiring a high number of face-to-face appointments.

## Supplemental Material

sj-docx-1-gph-10.1177_30502225251348292 – Supplemental material for Process Evaluation of a Randomized Controlled Trial With a Mobile Health Intervention for Children With Obesity Supplemental material, sj-docx-1-gph-10.1177_30502225251348292 for Process Evaluation of a Randomized Controlled Trial With a Mobile Health Intervention for Children With Obesity by Linnea Hedin, Maria Hagströmer, Claude Marcus and Pernilla Danielsson in Sage Open Pediatrics
