# Supplementary material for: Process Evaluation of a Randomized Controlled Trial With a Mobile Health Intervention for Children With Obesity
Source: Sage Open Pediatr. 2025 Jun 25;12:30502225251348292. doi: 10.1177/30502225251348292 (PMC12220870; doi:10.1177/30502225251348292)
Supplement: sj-docx-1-gph-10.1177_30502225251348292 – Supplemental material for Process Evaluation of a Randomized Controlled Trial With a Mobile Health Intervention for Children With Obesity [file sj-docx-1-gph-10.1177_30502225251348292.docx]

## Supplementary material

Supplementary Table 1. Baseline characteristics

| Baseline characteristics | Intervention n = 39 | Control n = 40 |
| --- | --- | --- |
| Girls, n (%) | 15 (38.5) | 22 (55) |
| Age years, mean (SD) [min–max] | 9.0 (2.0) [5.1–12.8] | 8.8 (2.3) [5.2–12.8] |
| BMI SDS baseline, mean (SD) [min–max] | 2.88 (0.42) [2.36–3.98] | 2.88 (0.37) [2.29–3.99] |
| Severe obesity, n (%) | 15 (38.5) | 22 (55) |

Supplementary Table 2. Recruitment, attrition, mHealth usage, and weight outcomes

| Recruitment & Attrition | Estimated | Outcome |
| --- | --- | --- |
| Recruitment intervention, n | 60 | 39 |
| Recruitment control n, | 60 | 40 |
| Attrition intervention, % | 30 | 44 |
| Attrition control, % | 30 | 20 |
| mHealth usage |  |  |
| Weakly weight frequency month 1, median (IQR) | 6.4 (2.7)^1^ | 3.5 (4.0)^2^ |
| Weakly weight frequency month 6, median (IQR) | 2.4 (3.0)^1^ | 1.6 (3.3)^3^ |
| Weakly weight frequency month 12, median (IQR) | - | 0.2 (0.8)^4^ |
| Change in BMI SDS |  |  |
| Δ BMI SDS 6 months intervention, mean (SD) | -0.23 (0.18)^1^ | -0.11 (0.21)^5^ |
| Δ BMI SDS 6 months control, mean (SD) | 0.01 (0.18)^1^ | -0.11 (0.25)^6^ |
| Δ BMI SDS 12 months intervention, mean (SD) | - | -0.12 (0.25)^7^ |
| Δ BMI SDS 12 months control, mean (SD) | - | -0.13 (0.26)^8^ |

^1^Data on estimated mHealth usage and change in BMI SDS stem from the feasibility trial by Johansson et al (2020)
Outcomes for weekly weights are based on participants remaining in the RCT; ^2^ n = 37; ^3^ n = 30; ^4^ n = 22
Outcomes for change in BMI SDS are based on observed data, i.e., participants remaining in the trial and attaining follow-up appointment ^5^ n = 25, ^6^ n = 31, ^7^ n = 22, ^8^ n = 32

Supplementary Table 3. NPT questions, results, and type of data for evaluating the trial

| NPT Component | Questions within the NPT framework | Process measures  Clinical staff | Results  Clinical staff | Process measures  Parents | Results  Parents | Type of data |
| --- | --- | --- | --- | --- | --- | --- |
| Coherence | Was the trial easy to describe? | - | Several staff members were guided on the eligibility criteria of previous treatment and on finding patients with obesity, using BORIS. Still, 5 individuals with overweight were offered participation—2 declined and 3 were excluded from analysis. | - | - | Researchers’ comments |
| Cognitive participation | Were target user groups prepared to invest time and energy in the trial? | Web-based questionnaires | Answer frequency:  14/14, 3 months  13/14, 6 months  10/14, 12 months | Web-based questionnaires  Attrition frequency and reasons | Answers based on participants remaining in the trial:  22/31, 3 months 20/28, 6 months 15/22, 12 months  Technical difficulties n=4  Disliking scale and/or activity monitor n=3  Lack of motivation n=2  Weighings stressful n=2  Problems with  mobile phone n=1  Not known n=4  Other reason n=1 | Answering frequency questionnaires  Attrition and reasons documented by staff |
| Collective action | Did the trial procedure promote or impede the work of user groups? | Recruitment  Comments from questionnaires | Difficulties with recruitment:  - High number of patients with neuropsychiatric disorders - High number of parents not speaking Swedish - Time-consuming finding participants with previous treatment and a BMI SDS change of ≤0.25 units  Three different digital system most likely obstructed the staff’s work:  *…Demanding with several systems…*  *It is one more system to keep track of…* | - | - | Self-reported data from staff on recruitment  Researchers’ comments  Open-ended questions |

Continuation, Supplementary Table 3. NPT questions, results, and type of data for evaluating the trial

| NPT Component | Questions within the NPT framework | Process measures  Clinical staff | Results  Clinical staff | Process measures  Parents | Results  Parents | Type of data |
| --- | --- | --- | --- | --- | --- | --- |
| Collective action | Did participation in the trial require extensive training for staff involved? |  | Two hours introduction on intervention and trial procedures. For several weeks before study enrollment, possibility to use the app, scale, and activity monitor to better understand the intervention. Encouraged to prepare by watching videos on the interface about downloading the app, creating weight loss target curves, and other trial procedures.  Additional introduction was offered, via telephone or face-to-face meetings, for staff that expressed a need for this. The trial coordinator had regular contact with each clinic throughout the whole trial |  |  | Researchers’ comments |
| Reflexive monitoring | Could participants contribute feedback about the trial once it was in ongoing? |  | Questionnaires at 3, 6 and 12 months  E-mail and telephone contact with trial coordinator |  | Questionnaires at 3, 6 and 12 months. Feedback to clinical staff | Researchers’ comments |

Supplementary Table 4. NPT questions, results, and type of data for evaluating the intervention

| NPT Component | Questions within the NPT framework | Study questionnaires & process measures Clinical staff | Results  Clinical staff | Study questionnaires & process measures  Parents/Children | Results  Parents/Children | Type of data |
| --- | --- | --- | --- | --- | --- | --- |
| Coherence | Was the intervention easy to describe? | Did you get a good explanation about the function and usage of the clinic’s interface? | 13/14 clinicians received a good explanation | Did you get a good explanation about the function and usage of the app? | 21/22 parents received a good explanation | Closed-ended questions at 3 months |
|  | Did the intervention have a clear purpose for all relevant participants? | What are the advantages with using the clinic’s interface?  What are the difficulties with using the clinic’s interface? | Providing distinct treatment goal:  8/14, 3 months 7/13, 6 months  5/10, 12 months  Difficult understanding weight loss target curves:  3/14, 3 months 1/13, 6 months 2/10, 12 months | Was the treatment goal clear to you?  Were you helped from the weight loss target curves in the app? | Treatment goal was clear to:  21/22, 3 months 20/20, 6 months  15/15, 12 months  Did not understand weight loss target curves:  1/22, 3 months 1/20, 6 months  1/15, 12 months | Closed--ended questions at 3, 6 and 12 months |
|  | What benefits did the intervention bring and to whom? | What are the advantages with using the clinic’s interface? | Facilitates communication with parents: 12/14, 3 months 10/13, 6 months 10/10, 12 months  Easily keep track of patient’s weight:  10/14, 3 months 9/13, 6 months 8/10, 12 months | Did the app help you to quickly get in touch with the staff?  What have been the greatest advantage with the app? | Completely/partly:  18/22, 3 months 16/20, 6 months 15/15, 12 months  Example:  *That there is a follow-up, which is not as charged for the child as an appointment to the doctor’s office. This with the weighings and the fact that we have got the diet right have made us get on the right track* | Closed--ended questions at 3, 6, and 12 months  Open-ended question at 3 months |
|  | Did the intervention fit with the overall goals and activity of the organization? | - | The intervention was in line with the national health care goals of extending digital health care | - | - | Researchers’ comments |

Continuation, Supplementary Table 4. NPT questions, results, and type of data for evaluating the intervention

| NPT Component | Questions within the NPT framework | Study questionnaires & process measures Clinical staff | Results  Clinical staff | Study questionnaires & process measures  Parents/Children | Results  Parents/Children | Type of data |
| --- | --- | --- | --- | --- | --- | --- |
| Coherence | Was the intervention clearly distinct from other interventions? | - | None of the clinics used digital interventions as a part of their standard care | - | None of the children in the trial was participating in another clinical trial | Researchers’ comments |
| Cognitive participation | Were target user groups likely to think the intervention was a good idea? | - | Most staff expressed positive attitudes about the intervention, but also concerns regarding required working time | - | According to staff, parents and children were mostly excited about the gamified activity monitor | Researchers’ comments |
|  | Were target user groups prepared to invest time and energy in the intervention? | How often did you, in general, log into the clinic’s interface?  Message frequency from staff to parents | <1 time/week:  3/14, 3 months 4/13, 6 months  2/10, 12 months  Clinic with the highest average of sent messages per patient* n=35  Clinic with the lowest average of sent messages per patient* n=7  *Independent of attrition | Weekly number of weights  Message frequency from parents to staff | First month, median (IQR) 3.5 (4.0) weights/week. Month 9 and forward median below 1.0 weight/week  Clinic with highest average of sent messages from parents* n=27  Clinic with lowest average of sent messages from parents* n=3  *Independent of attrition | Closed-ended questions at 3, 6 and 12 months  Objective data on weight- and message frequency during the whole trial |
| Collective action | Did the intervention promote or impede the work of user groups? | Required working time | Completers—Median (IQR) time (min), including documentation, spent on *appointments,* *phone calls* and *messages*; intervention 415 (203) vs control 240 (81), **p <.001**  Completers—Median (IQR) time (min), including documentation, spent on *appointments* and *phone calls*; intervention 250 (114) vs control 240 (81), p = .307 | - | - | Self-reported working time during the whole trial |

Continuation, Supplementary Table 4. NPT questions, results, and type of data for evaluating the intervention

| NPT component | Questions within the NPT framework | Study questionnaires & process measures Clinical staff | Results  Clinical staff | Study questionnaires & process measures  Parents/Children | Results  Parents/Children | Type of data |
| --- | --- | --- | --- | --- | --- | --- |
| Collective action | Did the intervention promote or impede the work of user groups? | Difficulties with the clinic’s interface?  What topics have you primarily addressed when sending messages? | Lack of message replies – time consuming:  *Makes it easier if the patient responds to messages, otherwise time consuming as you have to “chase” the family by phone*  Technical difficulties – time consuming  *A lot of technical problems with the activity-monitor, app and scale that takes a lot of time*  Most addressed topic was feedback on weight results, followed by reminders about weighings and using activity monitor | - | - | Closed- and open-ended questions at 3, 6 and 12 months |
|  | What effect did the intervention have on consultations? | - | Instructed to create a new weight loss target curve at each follow-up. No further instructions. To minimize interference in the staff’s treatment the intervention was deliberately designed to avoid telling staff what to do or say about obesity treatment in general | - | - | Researchers’ comments |
| Reflexive monitoring | How did users perceive the intervention once it had been in use for a while? | Difficulties and advantages with the clinic’s interface? | Technical difficulties the most common topic at 12 months:  *As there have been technical problems, eg wrist-band and transfer of activity, it has not been the positive reinforcement that it could have been*  At 12 months, 5/10 wanted to continue using the clinic’s interface in the future, and 5/10 were hesitant:  *More time and better technology is required* | What was it like with daily weighings?  Did your child find the activity monitor fun to use?  Benefits with the activity monitor? | Weighings were hard/very hard:  1/22, 3 months  5/20, 6 months  4/15, 12 months  Activity monitor fun:  18/22, 3 months 10/20, 6 months  Used monitor last 3months:  6/15, 12 months  *That the child becomes aware and motivated* | Closed- and open-ended questions at 3, 6 and 12 months |

Continuation, Supplementary Table 4. NPT questions, results, and type of data for evaluating the intervention

| NPT component | Questions within the NPT framework | Study questionnaires & process measures Clinical staff | Results  Clinical staff | Study questionnaires & process measures  Parents/Children | Results  Parents/Children | Type of data |
| --- | --- | --- | --- | --- | --- | --- |
| Reflexive monitoring | How did users perceive the intervention once it had been in use for a while? | - | - | Difficulties with the activity monitor?  Benefits with the app?  Difficulties with app? | 10/16 comments on poor quality or uncomfortable wristband.  Monitor stopped working, poor battery life, lack of game updates  5/16 comments on weighing and weight loss target curve being the most beneficial app-feature  3/16 comments on results from activity monitor as best app-feature  42 comments on app-difficulties throughout the trial – 32/42 about technical difficulties:   - Transfer data from activity monitor to the Provement-app - Transfer data from scale to the Provement-app - Log out and log in again to make the Provement-app work - High variation of registered weights   Other difficulties:  Lack of motivation  Set a routine  Measuring weight | Open-ended question at 3, 6 and 12 months |
